# The complete mitochondrial genome of *Trissolcus japonicus* (Hymenoptera: Scelionidae), the candidate for the biological control of *Halyomorpha halys* (Hemiptera: Pentatomidae)

**DOI:** 10.1080/23802359.2021.1948370

**Published:** 2021-07-09

**Authors:** Francesco Nardi, Claudio Cucini, Elena Cardaioli, Francesco Paoli, Giuseppino Sabbatini Peverieri, Pio Federico Roversi, Francesco Frati, Antonio Carapelli

**Affiliations:** a Department of Life Sciences, University of Siena, Siena, Italy; b CREA – Research Centre for Plant Protection and Certifications, Florence, Italy

**Keywords:** *Trissolcus japonicus*, *Halyomorpha halys*, mitochondrial genome, Platygastroidea

## Abstract

The samurai wasp *Trissolcus japonicus* (Ashmead, 1904) is a parasitoid hymenopteran that came into the limelight as the natural enemy of *Halyomorpha halys*. Here, we present the complete sequence of the mitochondrial genome of the CREATJ laboratory strain, naturally recovered in Italy in 2018. The molecule conforms to the typical model of animal mitochondrial genomes. Gene order is identical to that of its congeneric *Trissolcus basalis*. Phylogenetic analysis confirms its placement within monophyletic Scelionidae and Telenominae as the sister group of *T. basalis*.

*Trissolcus japonicus* (Ashmead, 1904) is an egg parasitoid and the natural enemy of *Halyomorpha halys.* Native to Asia, it followed its main host to the USA (Talamas et al. 2015), Switzerland (Stahl et al. 2019) and Northern Italy (Sabbatini Peverieri et al. 2018). Although multiple *Trissolcus* species can parasitize *H. halys* eggs (Talamas et al. 2019), *T. japonicus* was identified as the most promising candidate for biological control (Zhang et al. 2017). The strain CREATJ, used here, was established starting from females emerged from five egg masses of *H. halys* collected in 2018 in the area of Lodi (latitude 45.302793, longitude 9.478790) and used for permanent reared colonies at CREA facilities (Florence, Italy).

The complete mitochondrial genome of *T. japonicus* is likely to be of interest for biological control as it will: (a) allow to track the CREATJ strain in its natural spread in the field; and (b) allow the development of additional molecular markers to investigate intrageneric phylogenetic relationships and the invasion process of the species.

Total gDNA was extracted from a pool of individuals of the CREATJ strain using the QIAamp UCP DNA Micro Kit (QIAGEN, Hilden, Germany) and pooled with other unrelated species for sequencing (DNA voucher ID: CREATJ1, preserved in the Unisi-DSV collection, contact F.N., francesco.nardi@unisi.it; insect voucher ID: CREATJ1, preserved in the CREA collection, contact G.S.P, giuseppino.sabbatini@crea.gov.it). gDNA was sequenced at DNA LINK (Amsterdam, The Netherlands) using a TruSeq Nano DNA chemistry. Two different methods were used for sequence assembly: (a) MEGAHIT (version 1.2.9, default settings; Li et al. 2015); (b) NovoPlasty version 3.8.3 (default settings, *K* = 77, 101, 119; Dierckxsens et al. 2017) using sequence MT671804 as seed. Coverage was assessed in samtools version 1.11 (Li et al. 2009) after remapping in bbmap (kfilter = 22, subfilter = 15, maxindel = 80; sourceforge.net/projects/bbmap/). The resulting *T. japonicus* mitochondrial genome was automatically annotated using Mitos (version 1 (Bernt et al. 2013) and manually curated.

All complete, or semi-complete, mitochondrial genome sequences from Platygastroidea (10) were downloaded from GenBank, as well as representatives Proctotrupomorha (4), Evaniomorpha (1), and Ichneumonomorpha (1) as outgroups. Protein-coding gene sequences were processed through the EZmito webserver (Cucini et al. 2021). PartitionFinder version 2.1.1 (Lanfear et al. 2016) was used to identify optimal partitioning and models starting from partitions by strand/type/position, MrBayes version 3.2.7 (50 million generations, 25% burnin; Ronquist et al. 2012) was used for the phylogenetic analysis.

Sequencing produced a total of 186,490,629 read pairs. MEGAHIT produced >6 million contigs, one of which (16,410 bp, average coverage 1372, terminating with repeats at both ends) was identified as the candidate genome. NovoPlasty (*K* = 77 and 101) produced identical circularized candidate genomes (16,264 bp, average coverage ∼1300). Sequences differed by the presence of four imperfect tandem repeats (56–60 bp) in the latter corresponding to the boundaries of the former. The final submitted sequence corresponds to the NovoPlasty assembly (Supplementary Table 1). Coverage was unequal over the genome (Supplementary Figure 1), decreasing in areas characterized by strong secondary structures and high AT content, with a ∼2× spike encompassing tandem repeats. A short secondary sequence (MZ322407), closely related to *TrnC-TrnQ,* was recovered by Sanger sequencing and confirmed by remapping. Its minimal coverage (∼25) compared to the genome (∼1300) suggests its nuclear origin, although this has not been investigated further.

**Figure 1.**
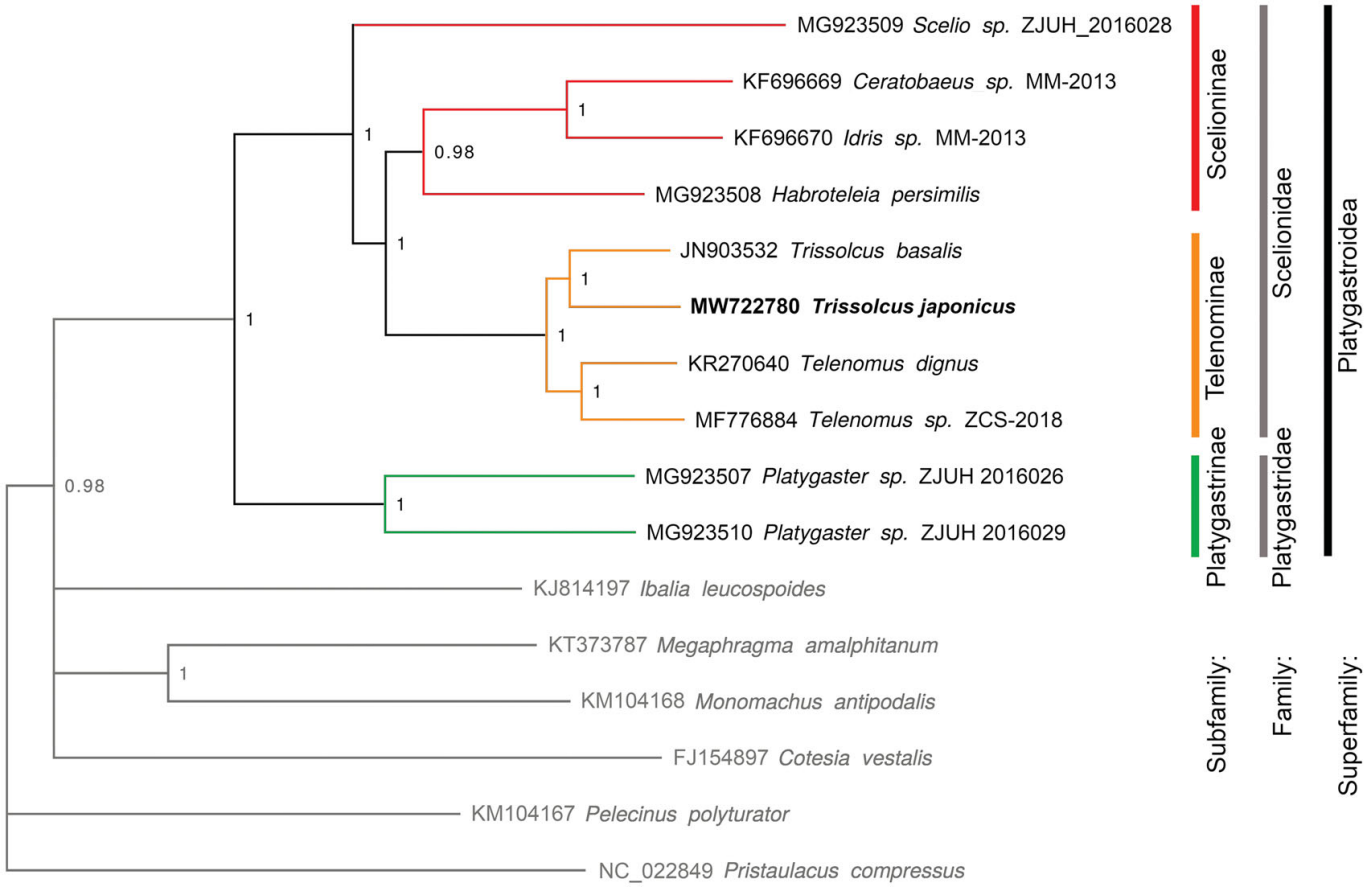
Phylogenetic placement of *T. japonicus* (in bold) in the context of Platygastroidea. Subfamilies are color coded and outgroup sequences appear in gray. Numbers at nodes represent posterior probability values and nodes with support <0.85 are collapsed.

The genome is a circular molecule of 16,264 bp. All canonical 37 genes are present and in the same order as in *Trissolcus basalis*. The *trnS1* and *trnR* lack the D-loop. The *trnR*, not annotated in *T. basalis*, was identified between *trnS1* and *nad5* and sequence similarity (52/54 sites) suggests its presence also in *T. basalis* (Mao et al. 2012) in partial overlap with *nad5*. Four imperfect tandem repeats of 56–60 bp were observed between *tnrC* and *trnY.* Coverage discontinuity suggests the possibility that the number of repeats may be larger or that some copy number variation is present in heteroplasmy (as in Nardi et al. 2001). Additional short imperfect repeats were observed in a low complexity area within the CR (nucleotides 15,300–15,600).

Limited to regions of sequence overlap, the genome presented here corresponds to the haplotype H1 in Sabbatini Peverieri et al. (2018), and is identical to sequences MT671799-804 (Zapponi et al. unpublished) sampled in Italy, as well as sequences MN615628 (Talamas et al. 2019), AB971832 (Mita et al. 2015), and MK188351/6 (Gariepy et al. unpublished) sampled in Japan. This indirectly supports that the Italian population originated from Japan, as suggested by Stahl et al. (2019). The phylogenetic analysis recovered well supported assemblages within the ingroup (Figure 1). *T. japonicus* clusters with the congeneric *T. basalis*. The two families Platygastridae and Scelionidae are recovered as monophyletic. Within Scelionidae, subfamily Telenominae was recovered as monophyletic while Scelioninae appeared non-monophyletic due to the position of *Scelio sp*. Phylogenetic relationships, limited to shared sequences, are in line with Shen et al. (2019) and Tang et al. (2019), including the non-monophyly of Scelioninae in the latter.

## Data Availability

Data supporting the findings of this study are openly available in NCBI at https://www.ncbi.nlm.nih.gov/ under BioProject ID PRJNA715606, BioSample accession SAMN18354450, SRA accession SRR14001126 and nucleotide accession MW722780 (annotated genome).
